# Modeling of pulmonary deposition of agents of open and fixed dose triple combination therapies through two different low-resistance inhalers in COPD: a pilot study

**DOI:** 10.3389/fmed.2023.1065072

**Published:** 2023-05-05

**Authors:** Tamas Erdelyi, Zsofia Lazar, Árpád Farkas, Peter Furi, Attila Nagy, Veronika Müller

**Affiliations:** ^1^Department of Pulmonology, Semmelweis University, Budapest, Hungary; ^2^Environmental Physics Department, Centre for Energy Research, Budapest, Hungary; ^3^Department of Applied and Nonlinear Optics, Wigner Research Centre for Physics, Budapest, Hungary

**Keywords:** COPD, fixed dose triple therapy, open triple therapy, deposition, repeatability, modeling

## Abstract

**Introduction:**

Inhalation therapy is a cornerstone of treating patients with chronic obstructive pulmonary disease (COPD). Inhaler devices might influence the effectiveness of inhalation therapy. We aimed to model and compare the deposition of acting agents of an open and a fixed dose combination (FDC) triple therapy and examine their repeatability.

**Methods:**

We recruited control subjects (Controls, *n* = 17) and patients with stable COPD (S-COPD, *n* = 13) and those during an acute exacerbation (AE-COPD, *n* = 12). Standard spirometry was followed by through-device inhalation maneuvers using a pressurized metered dose inhaler (pMDI) and a soft mist inhaler (SMI) to calculate deposition of fixed dose and open triple combination therapies by numerical modeling. Through-device inspiratory vital capacity (IVC_d_) and peak inspiratory flow (PIF_d_), as well as inhalation time (t_in_) and breath hold time (tbh) were used to calculate pulmonary (PD) and extrathoracic deposition (ETD) values. Deposition was calculated from two different inhalation maneuvers.

**Results:**

There was no difference in forced expiratory volume in 1 s (FEV1) between patients (S-COPD: 42 ± 5% vs. AE-COPD: 35 ± 5% predicted). Spiriva^®^ Respimat^®^ showed significantly higher PD and lower ETD values in all COPD patients and Controls compared with the two pMDIs. For Foster^®^ pMDI and Trimbow^®^ pMDI similar PD were observed in Controls, while ETD between Controls and AE-COPD patients did significantly differ. There was no difference between COPD groups regarding the repeatability of calculated deposition values. Ranking the different inhalers by differences between the two deposition values calculated from separate maneuvers, Respimat^®^ produced the smallest inter-measurement differences for PD.

**Discussion:**

Our study is the first to model and compare PD using pMDIs and an SMI as triple combination in COPD. In conclusion, switching from FDC to open triple therapy in cases when adherence to devices is maintanined may contribute to better therapeutic effectiveness in individual cases using low resistance inhalers.

## 1. Introduction

Chronic obstructive pulmonary disease (COPD) affects more than 380 million people worldwide (1). Inhalation therapy is a cornerstone of the treatment of airway diseases, including high burden diseases as COPD and asthma (1, 2). According to international guidelines the inhalation therapy of COPD patients with severe obstructive ventilatory disorder, persistent symptoms and frequent exacerbations (Global Initiative for Chronic Obstructive Lung Disease: GOLD D category in most of our cases) contains long-acting beta2-agonist (LABA) and muscarinic antagonists (LAMA) as bronchodilators and inhaled corticosteroids (ICS) especially in patients with exacerbations and high blood eosinophil count (1). Many commercially available inhalers are a combination of ICS-LABA, LABA-LAMA, or fixed triple combination (ICS-LABA-LAMA; FDC). Despite the availability of triple combinations, many patients use two different devices, mainly combination of ICS-LABA and a mono-LAMA inhaler (3).

The effectivity of inhalation therapy has numerous influencing factors which are connected to the drug, the device or the patient. The largest variety of devices belong to dry powder inhalers (DPI) and many combinations are commercially available including triple FDC. Pressurized metered dose inhaler (pMDI) has a low number of different drug combinations including triple FDC and ICS-LABA, while soft mist inhaler (SMI) is only represented by the device Respimat® and is containing LABA-LAMA or LAMA monotherapy.

Various studies examined the effectiveness of inhalation therapy *in vivo*. Additionally *in vitro* and *in silico* investigations measured the deposition rate of the emitted dose (4, 5). *In vivo* studies mostly apply radioscintigraphy and can be complicated upon repeated measurements as it imposes a burden of radiation on the subjects (6, 7). However, other studies applied pharmacokinetic methods to measure pulmonary deposition of inhaled particles by measuring serum levels and urinary excretion of specific agents (8–10). Nonetheless, pharmacokinetic methods are not able to differentiate between the deposition into different regions of the lung and it is not capable to reveal the amount of drug removed by mucociliary clearance (11). *In vitro* measurements only require the equipment to produce a replica of the airways and is limited by the natural variety of the different subjects’ anatomy (5). *In silico* studies such as computational fluid dynamic simulations or numerical simulations (e.g., the Stochastic Lung Model) have the benefit of repeated measurements not requiring personal and material input but they need a validation by *in vivo* models before usage (12–14).

As the device-handling plays a critical role in the success of inhalation therapy, many factors influencing patient conduct can be investigated (15–17). Sufficient peak inspiratory flow (PIF) is crucial using DPI devices, which might be difficult to generate for patients with severe COPD (18). Appropriate handling of pMDI and SMI devices demand the precision from patients regarding the timing of actuation and inspiration, adequate breath-hold time, correct posture and device position, and sufficient inspiratory volumes (16). There are many ways to observe the accuracy of device handling, but limited number of publications focuses on assessing uniformity of inhalation maneuvers through these devices known as repeatability resulting in predictable PD every day.

Our aim was to investigate inhalation maneuvers through commercially available pMDI and SMI devices, and assess deposition repeatability of FDC or open triple therapy in severe GOLD D COPD patients by numerical modeling.

## 2. Materials and methods

### 2.1. Subjects

Patients with stable COPD (S-COPD, *n* = 13) were recruited during regular outpatient visits, patients with exacerbated COPD (AE-COPD, *n* = 12) were included <72 h after hospital admission due to an acute severe relapse. Patients had been previously diagnosed with COPD by a respiratory specialist according to 2015 Global Initiative for Chronic Obstructive Lung Disease (GOLD) as post-bronchodilator FEV_1_/FVC < 0.70 (19). Therapy was decided by the treating physician, but all patients with AE were treated with systemic steroids. Control volunteers (Control, *n* = 17) did not have a chronic respiratory disease and were recruited from employees of the Department of Pulmonology, Semmelweis University, Budapest, Hungary. Individuals in the S-COPD and Control groups with acute respiratory tract infections within 2 weeks and AE-COPD group who suffered from pneumonia or needed non-invasive or invasive ventilation were excluded. Subjects were recruited between April and December 2015.

All procedures were performed in accordance with the 1964 Helsinki declaration and its later amendments or comparable ethical standards. All subjects were informed about the methods and aims of the measurements and signed the informed consent form. The study was approved by the Semmelweis University Regional and Institutional Committee of Science and Research Ethics (SE TUKEB 239/2015).

### 2.2. Study design

The subjects attended a single visit which was followed by data processing and numerical modeling. All patients performed standard lung function tests, which were followed by the two consecutive inhalation maneuvers per device using two different inhalers after a minimum of a 30-min break. Subjects filled out disease-specific and generic quality of life questionnaires. Subsequently, the deposition calculations were performed by the Stochastic Lung Model (13).

### 2.3. Lung function measurements

Electronic spirometer and body plethysmography (PDD-301/s, Piston, Budapest, Hungary) were used for lung function measurements performed according to the European Respiratory Society (20, 21). None of the records were post-bronchodilator measurements.

### 2.4. Inhalation maneuvers through different inhalers

Commercially available pressurized metered-dose inhaler (pMDI inhalation solution placebo for Foster®/Trimbow®) and soft mist inhaler (placebo for Respimat®) were used.

For through-device lung function testing electronic spirometer was used (PDD-301/sh, Piston, Budapest, Hungary), which has built-in ambient temperature, pressure and humidity sensors for the fully automatic BTPS correction, as described in detail in our previous study (22). The spirometer is equipped with a PinkFlow flowmeter (PPF-18, Piston, Budapest, Hungary), which measures flow based on the principle of a symmetric and averaging Pitot tube, and was connected directly to the pMDI or SMI. Subjects were instructed for 5–10 min before the measurements to explain and correct inhalation maneuvers as recommended by the manufacturers of each device. Steps of the inhalation maneuver included: (1) preparation of the device, (2) long exhalation, (3) attachment of the inhaler to the flexible connecting piece, (4) deep inhalation through the inhaler to total lung capacity, with optimal actuation of pMDI and SMI by the examiner and simultaneous recording of the pre-specified parameters, (5) breath-holding for 10 s (when possible) while the inhaler device was detached from the connecting piece; and (6) long exhalation. Through-device inspiratory vital capacity (IVCd) and peak inspiratory flow (PIF_d_), inhalation time (t_in_) and breath-hold time (t_bh_) were recorded. Measurements for both pMDI and Respimat® were performed and randomly followed after at least 5-min break by the second sequence of maneuvers in all patients and controls.

### 2.5. Assessment of symptoms and quality of life

Subjects filled out the Modified Medical Research Council (mMRC) and the Hungarian version of the COPD Assessment Test (CAT), and the Visual Analogue Scale (VAS) scaled from 0 to 10, to measure the general health condition of the participants.

### 2.6. Numerical modeling of pulmonary and extrathoracic deposition

Pulmonary (PD) and extrathoracic (ETD) deposition fraction values were calculated as a percent of the metered dose using the Stochastic Lung Model (SLM). The model was primarily developed by Koblinger and Hofmann and afterwards it has undergone further development. The model has been validated and used to simulate the pulmonary deposition of different aerosols as well as inhaled drug particles (13, 14, 23). In the SLM model the structure of the conducting airways is built up stochastically based on distribution functions of airway lengths, diameters, branching angles and gravity angles Raabe (24). The geometry of the acinar airways is built up based on the description of Haefeli-Bleuer and Weibel (25). The model is calculating deposition fractions in the extrathoracic airways based on empirical deposition formulas. In the pulmonary airways deposition fractions are computed by tracking large numbers of inhaled particles after their inhalation until they deposit in the airways or leave the lungs *via* exhalation. In the model the particles can deposit due to impaction, gravitational settling and Brownian diffusion. As input data the breathing parameters and the size distribution and density of the drug particles need to be provided. The inhalation parameters are the standard spirometry and body plethysmography measurement results, such as residual volume (RV) and through-device spirometry data, such as IVC, T_in_ and T_bh_, which were provided for both pMDI and Respimat® devices. For the calculation we used the particle size distribution values of Spiriva® Respimat®, Foster® pMDI and Trimbow® pMDI. PD and ETD values were calculated from the first and second inhalation maneuver and their mean was used for further statistical analysis.

### 2.7. Statistical analysis

Statistical analysis was performed using GraphPad Prism software 8 (GraphPad Software, La Jolla, CA, United States) and SPSS Statistics V22 (International Business Machines Corporation, NY, United States). The results are expressed as the mean ± standard error of the mean (SEM) or median (interquartile range). One-way ANOVA followed by Bonferroni’s multiple comparison test or Kruskal-Wallis test with Dunn’s multiple comparison test were used as appropriate. Repeatability of deposition values was assessed by the Bland–Altman test (26). Results were considered to be statistically significant when the *p* value was less than 0.05.

## 3. Results

### 3.1. Clinical characteristics of participants

Patient and control volunteer characteristics are summarized in Table 1. COPD patients were significantly older, more often smokers and had higher cumulative smoking impact. All AE-COPD patients fulfilled the criteria of GOLD D category. COPD patients had a high number of comorbidities but there were no significant differences between stable and exacerbated patients in this regard. Patients with exacerbations were more symptomatic using mMRC, CAT and VAS scores. The maintenance inhalation therapy was similar between patient groups, most patients being on triple therapy.

**Table 1:** Clinical characteristics of controls and patients.

	Control	S-COPD	AE-COPD
Number (*n*)	17	13	12
Female/male	10/7	9/4	9/3
Age (years)	43 ± 4	65 ± 2*	61 ± 2*
BMI (kg/m^2^)	25.0 ± 0.9	25.6 ± 1.4	27.1 ± 2.0
Smoking habit, *n* (%)^**^			
Current smoker	8 (47)	4 (31)	7 (58)
Former smoker	1 (6)	9 (69)	5 (42)
Never smoker	8 (47)	0 (0)	0 (0)
Pack years	18 ± 5	50 ± 5*	36 ± 3*
GOLD category 2017, *n* (%)			
A	NA	1 (8)	0 (0)
B	NA	1 (8)	0 (0)
C	NA	5 (38)	0 (0)
D	NA	6 (46)	12 (100)
Quality of life			
mMRC	0 (0–0)^a^	**2 (1–2)** ^b^	4 (3–4)*
CAT	2 (0–6)	**11 (7–22)**^b^,*	27 (18–30)*
VAS	1 (0–3)^c^	**5 (4–5)**^b^,*	**8 (7–10)**^d^,*
Comorbidities, *n* (%)			
Osteoporosis	NA	0 (0)	3 (25)
Diabetes mellitus	NA	1 (8)	3 (25)
Hypertension	NA	4 (31)	2 (17)
Atherosclerosis	NA	4 (31)	4 (33)
Myocardial infarction	NA	0 (0)	2 (17)
Stroke	NA	0 (0)	2 (17)
Maintenance COPD therapy, *n* (%)			
ICS	NA	9 (69)	12 (100)
LABA	NA	12 (92)	12 (100)
LAMA	NA	12 (92)	12 (100)
Theophylline	NA	3 (23)	6 (50)

*p* < 0.05 vs. Control, ^**^ Chi-square test: *p* < 0.01. Significant differences are highlighted in bold. BMI, Body Mass Index; CAT, COPD Assessment Test; AE-COPD: patients with exacerbated COPD; GOLD, Global Initiative for Chronic Obstructive Lung Disease; ICS, inhaled corticosteroid; LABA, long-acting beta_2_-agonist; LAMA, long-acting muscarinic antagonist; mMRC, modified Medical Research Council; NA, not applicable; S-COPD: patients with stable COPD; VAS, Visual Analogue Scale. Data are shown as mean ± standard error of the mean (SEM) or median (interquartile range).

a *n* = 16.

b *n* = 10.

c *n* = 15.

d *n* = 11.

### 3.2. Lung function results

Lung function parameters revealed similarly severe airflow obstruction and lung hyperinflation in both COPD groups, while normal lung function parameters were noted in the Control group (Table 2).

**Table 2:** Lung function values.

	Control	S-COPD	AE-COPD
Number (*n*)	17	13	12
FVC, % predicted	102 ± 3	79 ± 6*	67 ± 7*
FEV_1_, % predicted	95 ± 2	42 ± 5*	35 ± 5*
FEV_1_/FVC, %	79 ± 2	44 ± 3*	49 ± 3*
PEF, % predicted	85 ± 8	41 ± 4*,^a^	34 ± 3*,^b^
FEF_25-75%_, % predicted	76 ± 5	18 ± 3*	17 ± 2*
PIF, L/s	5 ± 0.4	2 ± 0.1*	3 ± 0.3*
IVC, % predicted	99 ± 3	77 ± 5*	67 ± 5*
TLC, % predicted	93 ± 2	103 ± 5*	113 ± 8*
TGV, % predicted	119 ± 5	168 ± 11*	193 ± 15*
RV, % predicted	83 ± 6	152 ± 15*	192 ± 19*
RV/TLC	0.28 ± 0.02	0.56 ± 0.03*	0.66 ± 0.04*
Raw, % predicted	108 ± 6	**295 ± 25** ^*a^	297 ± 31*

^*^p < 0.05 vs. Control, Significant differences are highlighted in bold. AE-COPD: patients with exacerbated COPD; FEF_25-75%_, forced expiratory flow at 25–75% of the pulmonary volume; FEV_1_, forced expiratory volume in the 1st second; FVC, forced vital capacity; IVC, inspiratory vital capacity; PEF, peak expiratory flow; PIF, peak inspiratory flow; Raw, airway resistance; RV, residual volume; S-COPD: patients with stable COPD; TGV, thoracic gas volume; TLC, total lung capacity. Data are shown as mean ± standard error of the mean (SEM).

a *n* = 12.

b *n* = 13.

### 3.3. Through-device inhalation parameters using different inhalers

IVC_d_, PIF_d_, t_in_ and t_bh_ were tested for pMDI and SMI devices (Table 3). IVC_d_ was lower as measured through both devices than during normal spirometry in controls, while only slightly lower in both COPD groups. In the Control and both COPD groups PIF_d_ was significantly lower as compared to PIF during spirometry for both devices. Inhalation time (t_in_) was on average between 2–3 s for all groups. Mean t_bh_ was above 10 s in Controls and S-COPD and significantly lower in AE-COPD patients compared to S-COPD patients.

**Table 3:** Spirometric and inhalation parameters measured through the different inhalers.

Control group (*n* = 17)		
Spirometry	IVC (L)	4.02 ± 0.26
Spirometry	PIF (L/s)	5.08 ± 0.36
	pMDI	Respimat®
IVC_d_ (L)	**3.36 ± 0.22** ^*^	**3.61 ± 0.21** ^*^
PIF_d_ (L/s)	**2.61 ± 0.22** ^*^	**2.19 ± 0.15** ^*^
t_in_ (s)	2.23 ± 0.22	2.51 ± 0.23
t_bh_ (s)	9.95 ± 0.12	9.93 ± 0.16
S-COPD group (*n* = 13)		
Spirometry	IVC (L)	**2.35 ± 0.2** ^**^
Spirometry	PIF (L/s)	**2.48 ± 0.15** ^**^
	pMDI	Respimat®
IVC_d_ (L)	**2.23 ± 0.17** ^**^	**2.29 ± 0.21** ^**^
PIF_d_ (L/s)	**1.80 ± 0.16** ^*,**^	**1.48 ± 0.14** ^*,**^
t_in_ (s)	2.44 ± 0.26	2.57 ± 0.27
t_bh_ (s)	10.39 ± 0.1	10.57 ± 0.18
AE-COPD group (*n* = 12)		
Spirometry	IVC (L)	**2.17 ± 0.25** ^**^
Spirometry	PIF (L/s)	**2.80 ± 0.32** ^**^
	pMDI	Respimat®
IVC_d_ (L)	**2.06 ± 0.23** ^**^	**2.18 ± 0.21** ^**^
PIF_d_ (L/s)	**1.79 ± 0.13** ^*,**^	**1.48 ± 0.12** ^*,**^
t_in_ (s)	2.3 ± 0.28	2.52 ± 0.26
t_bh_ (s)	**9.55 ± 0.16** ^***^	**9.44 ± 0.40** ^***^

IVC, inspiratory vital capacity; PIF, peak inspiratory flow; IVC_d_, through-device inspiratory vital capacity; PIF_d_, through-device peak inspiratory flow; t_in_, inhalation time; t_bh_, breath hold time; ^*^p < 0.05 vs. values obtained by standard spirometry; ^**^p < 0.05 vs. Control group; ^***^p < 0.05 vs. S-COPD. Significant differences are highlighted in bold. Data are shown as mean ± standard error of the mean (SEM).

### 3.4. Pulmonary (PD) and extrathoracic deposition (ETD)

**Figure 1:**
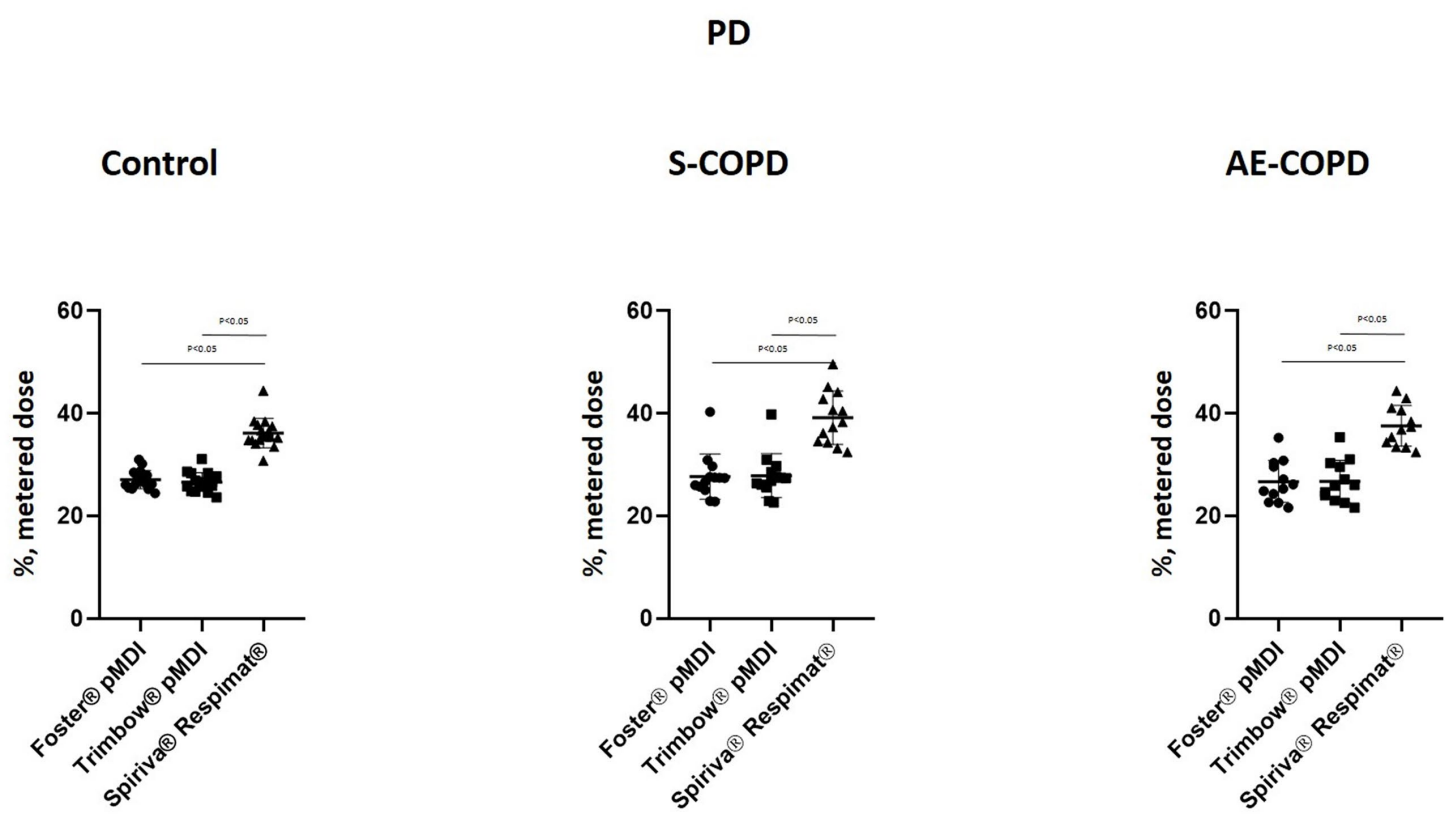
Pulmonary deposition (PD). AE-COPD: patients with exacerbated COPD; S-COPD: patients with stable COPD.

**Figure 2:**
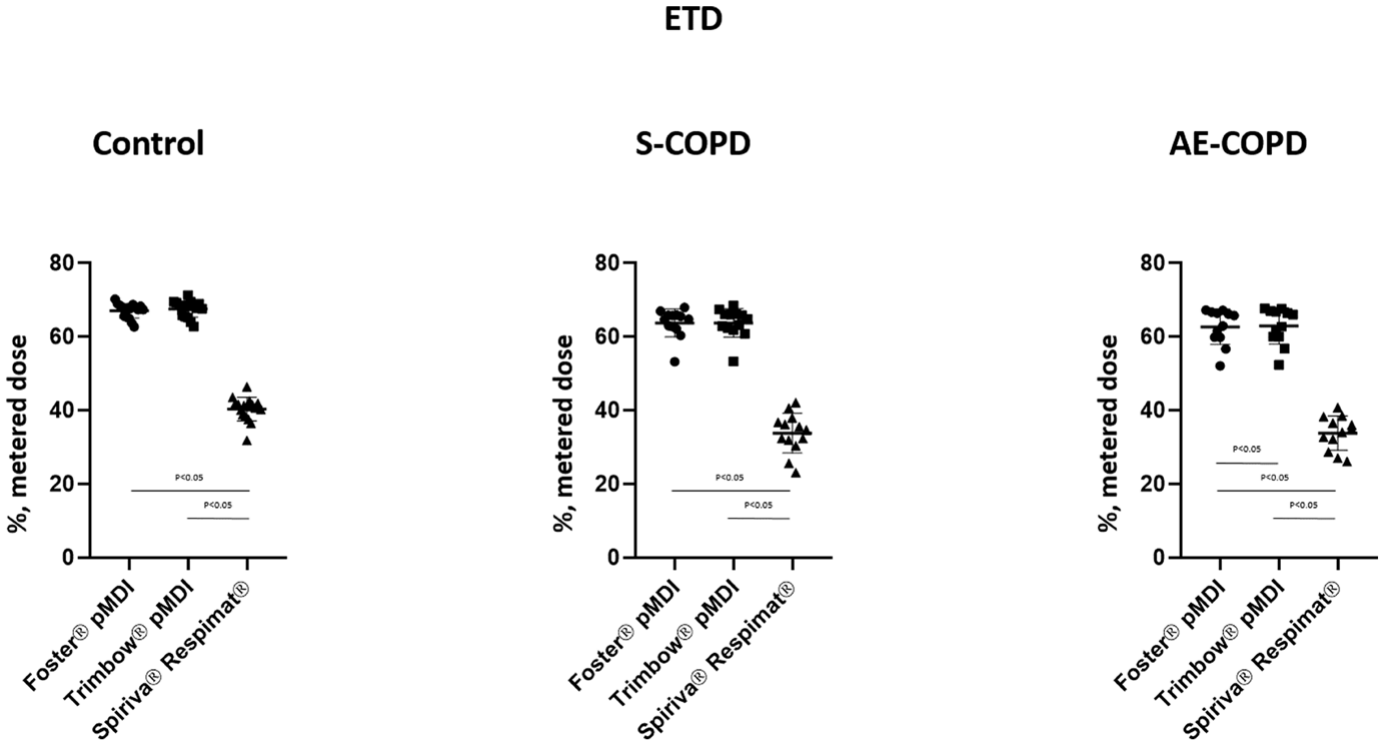
Extrathoracic deposition (ETD). AE-COPD: patients with exacerbated COPD; S-COPD: patients with stable COPD.

The results of numerical modeling for Foster® pMDI, Trimbow® pMDI and Spiriva® Respimat® are summarized in Figures 1, 2. Both COPD groups and Controls showed significant difference by Spiriva® Respimat® compared to the two pMDIs regarding PD and ETD. Spiriva® Respimat® produced much higher PD than Foster® pMDI and Trimbow® pMDI. For Foster® pMDI and Trimbow® pMDI similar PD were observed in Controls, while ETD between Controls and AE-COPD patients did significantly differ. ETD values were significantly lower in all COPD patients compared to heathy volunteers. Spiriva® Respimat® showed significantly lower ETD values than Foster® pMDI and Trimbow® pMDI.

**Table 4:** Repeatability of lung and extrathoracic deposition values calculated from repeated measurements.

	*n*	Bias	*p*	95% LoA	CR
	**C**				
Lung					
Foster® pMDI	17	0.80	**0.02** ^*^	−1.68 – 3.28	2.87
Trimbow® pMDI	17	0.70	**0.005** ^*^	−1.21 – 2.61	2.31
Spiriva® Respimat®	17	1.19	0.13	−4.86 – 7.23	6.31
ET					
Foster® pMDI	17	−0.5	**0.046** ^*^	−2.35 – 1.36	2.05
Trimbow® pMDI	17	−0.59	**0.032** ^*^	−2.62 – 1.44	2.29
Spiriva® Respimat®	17	−1.35	0.12	−7.93 – 5.22	6.91
	**S-COPD**				
Lung					
Foster® pMDI	13	−0.89	0.42	−8.45 – 6.67	7.47
Trimbow® pMDI	13	−1.62	0.38	−14.2 – 10.91	12.46
Spiriva® Respimat®	13	0.25	0.83	−7.89 – 8.39	7.84
ET					
Foster® pMDI	13	0.89	0.46	−7.32 – 9.11	8.08
Trimbow® pMDI	13	1.31	0.26	−6.46 – 9.08	7.9
Spiriva® Respimat®	13	−1.17	0.51	−13.28 – 10.94	11.86
	**AE-COPD**				
Lung					
Foster® pMDI	12	1.91	0.23	−8.24 – 12.06	10.42
Trimbow® pMDI	12	1.8	0.27	−8.78 – 12.38	10.72
Spiriva® Respimat®	12	0.72	0.58	−7.77 – 9.21	8.25
ET					
Foster® pMDI	12	−2.06	0.24	−13.22 – 9.1	11.42
Trimbow® pMDI	12	−2.07	0.25	−13.57 – 9.43	11.74
Spiriva® Respimat®	12	−0.93	0.52	−10.34 – 8.47	9.19

* *p*-Value for one-sample *t*-test of the bias.

AE-COPD: patients with exacerbated COPD; CR, coefficients of repeatability; LoA, Bland–Altman 95% limits of agreement; n, number of subjects; S-COPD: patients with stable COPD. Significant differences are highlighted in bold.

### 3.5. Repeatability of pulmonary (PD) and extrathoracic deposition (ETD) values calculated from repeated measurements

**Figure 3:**
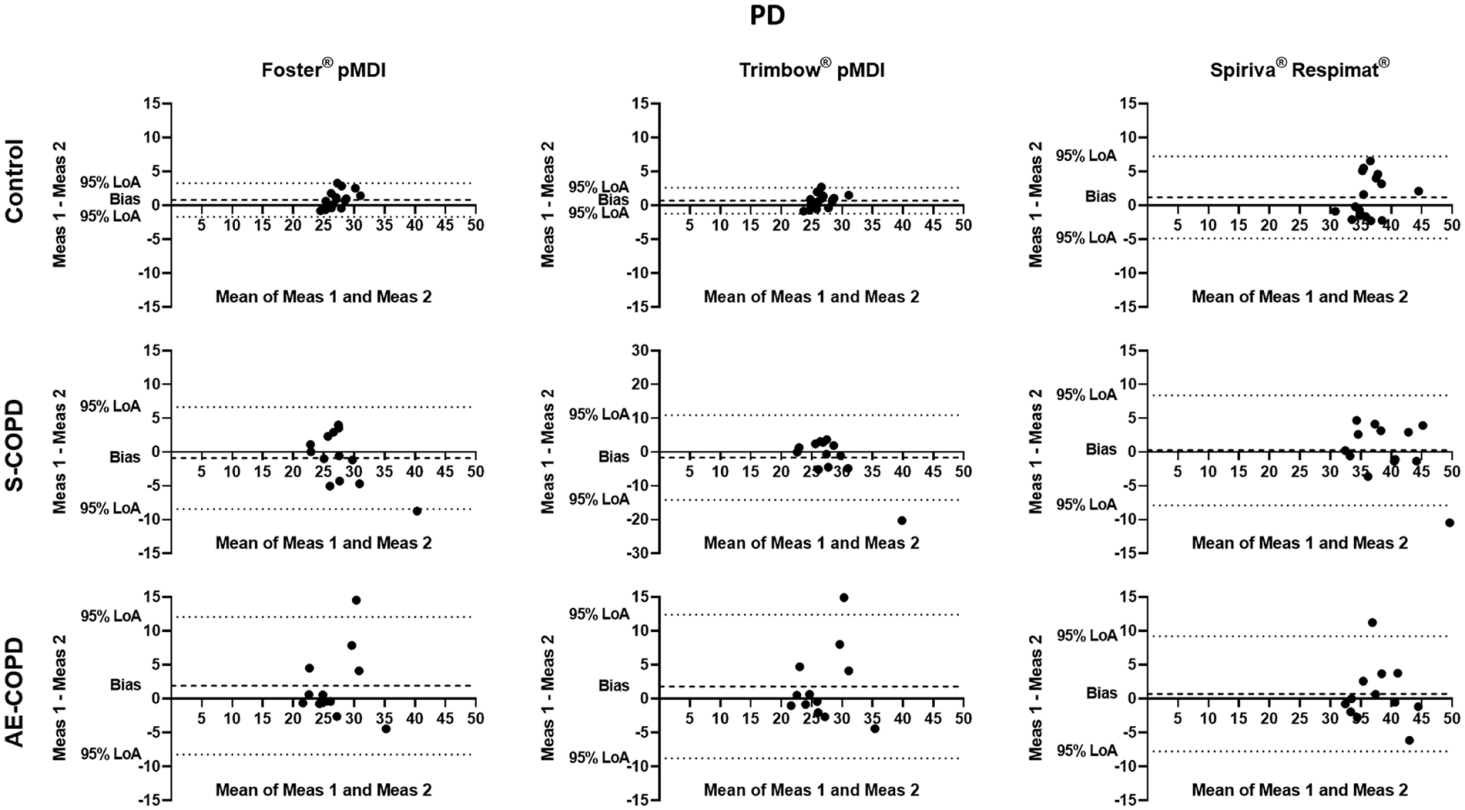
Bland–Altman analysis of pulmonary deposition (PD). The X-axis represents the mean of the two measurements for PD, while the Y-axis shows the difference of the repeated measurements (first measurement–second measurement). Each dot represents a person. The dashed line shows the average of the difference for all subjects. AE-COPD: patients with exacerbated COPD; LoA, Bland–Altman 95% limits of agreement; Meas, measurement; S-COPD: patients with stable COPD.

**Figure 4:**
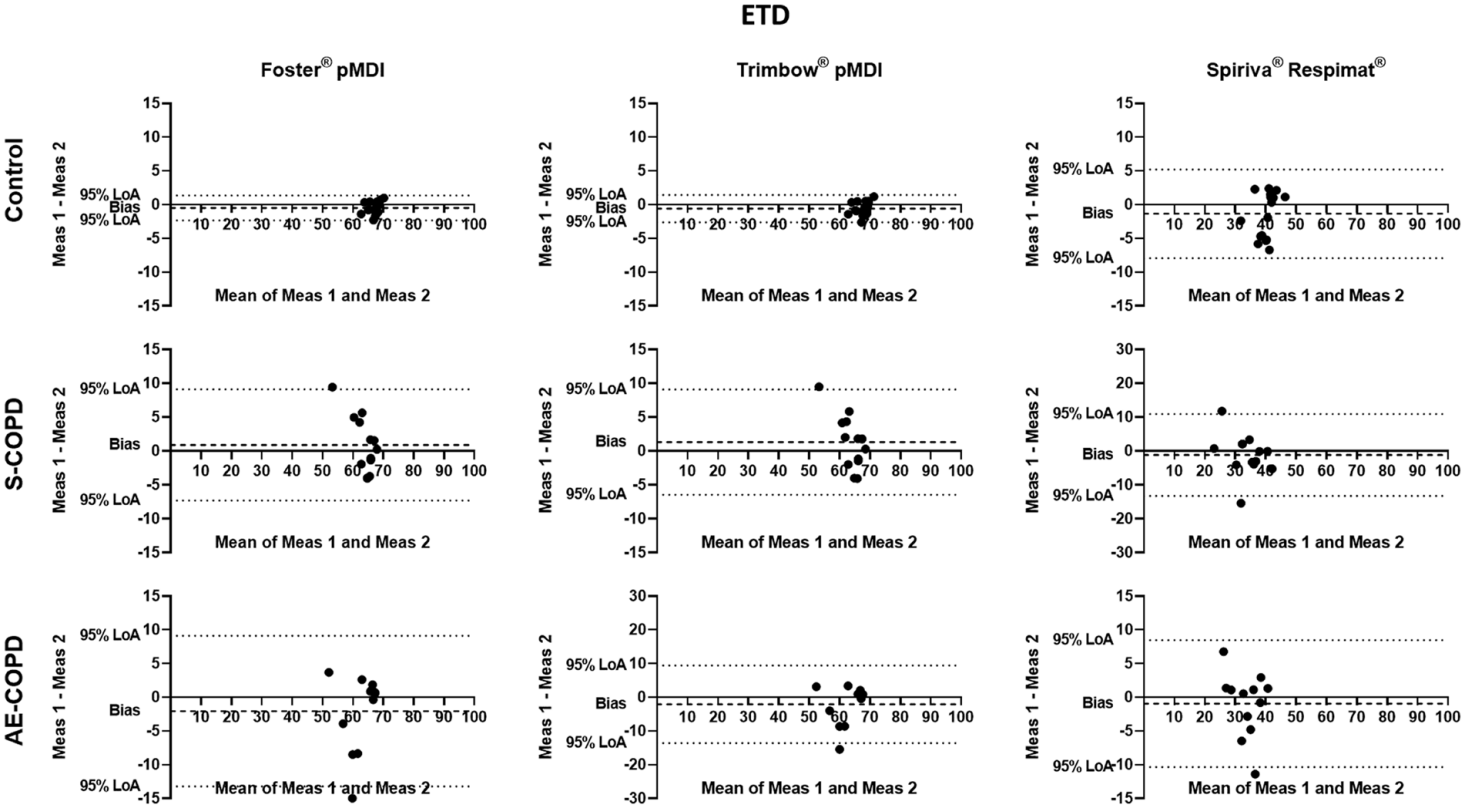
Bland–Altman analysis of extrathoracic deposition (ETD). The X-axis represents the mean of the two measurements for ETD, while the Y-axis shows the difference of the repeated measurements (first measurement–second measurement). Each dot represents a person. The dashed line shows the average of the difference for all subjects. AE-COPD: patients with exacerbated COPD; LoA, Bland–Altman 95% limits of agreement; Meas, measurement; S-COPD: patients with stable COPD.

The Bland–Altman analysis was used to define the variability of the PD and ETD values calculated from inhalation maneuver parameters through a given inhaler and the corresponding particle size distribution. Significant individual differences were present in all tested medications regarding PD and ETD (Figures 3, 4). The X-axis represents the mean of the two calculations for deposition values, while the Y-axis shows the difference of the two calculated values from repeated measurements (1^st^ measurement–2^nd^ measurement).

We also calculated the bias (difference between the X-axis and the average mean of the two calculations for all subjects) for PD and ETD in Control, S-COPD and AE-COPD groups for each inhaler. We found that PD was significantly higher by the values calculated from second measurements in Controls using Foster® pMDI and Trimbow® pMDI. There was a tendency in healthy volunteers by Spiriva® Respimat® for the second value to be higher. There was no difference between the two values in either COPD group regarding the two pMDI devices but in S-COPD patients the second value tended to be lower while in AE-COPD patients higher.

The 95% limits of agreement and the coefficients of repeatability (CR) of PD and ETD through the different inhalers were high and variable in both, controls and patients It is important to highlight that low CR represents better repeatability. Of note, the CR in S-COPD and AE-COPD patients for PD was the largest using Trimbow® pMDI (see Table 4).

### 3.6. Ranking of inhalers based on the differences between deposition values

**Figure 5:**
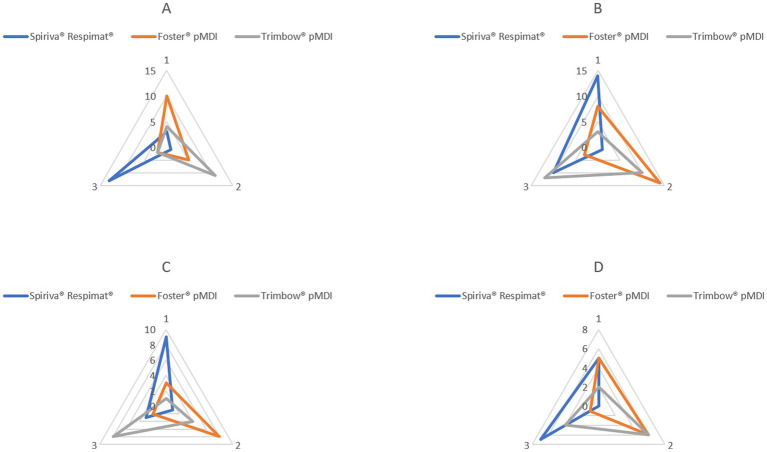
Repeatability sequence summary for the three inhalers regarding PD. **(A)** Control: healthy volunteers (*n* = 17); **(B)** All-COPD: patients with stable and exacerbated COPD (*n* = 25); **(C)** S-COPD: patients with stable COPD (*n* = 13); **(D)** AE-COPD: patients with exacerbated COPD (*n* = 12). By each control subject and patient, a rank number between 1 and 3 was associated with each inhaler regarding the magnitude of the difference between the two values for PD, respectively. Rank 1 was given to the device with the lowest difference between the two inspiratory measurements followed by Rank 2 and 3. On the figure, rank numbers are shown by three edges of the axes, and the sum of subjects with a certain rank is indicated on the axes. COPD: chronic obstructive pulmonary disease.

Ranking the three inhalers based on the differences between the two values of PD is shown in Figure 5. This highlights the inhalers with the smallest difference (given Rank 1 followed by Rank 2 and Rank 3) in values between the two deposition results. Similarly, to the findings based on the CR values in patients with COPD, Respimat® produced the smallest inter-measurement differences for PD.

## 4. Discussion

Our study compared commercially available pMDI and SMI devices for PD repeatability of FDC (ICS-LABA-LAMA) and open triple (ICS-LABA pMDI and LAMA SMI) therapy in severe COPD patients. Numerical modeling of PD provided ~25–28% pulmonary drug deposition in our model for pMDI and ~ 36–39% for SMI. Very similar results were shown in gamma scintigraphy studies using the active agents of Trimbow® pMDI in healthy and asthmatic subjects and ~ 37% using Respimat® SMI in untrained COPD patients validating our *in silico* method (7, 27).

According to previous scintigraphy studies, in asthmatic patients with mild airway obstruction there was no difference in pulmonary drug deposition using Trimbow® pMDI as compared to healthy controls (7). In contrast, we examined COPD patients with severe airway obstruction, where no scintigraphy data are available to assess PD using two triple combination regimens. Notwithstanding our model cannot separate drug deposition in central and peripheral regions of the lung as gamma scintigraphy, on the other hand it is safe and reproducible. We were able to evaluate deposition values in the lung and extrathoracically and compare drug delivery of FDC and open triple therapies in COPD patients in stable state and during AE. One of the main findings is that there was no difference in PD between S-COPD and AE COPD patients for both low resistance pMDI and SMI devices. Besides the high reproducibility of numerical modeling, we put a lot of emphasis on the repeatability of inhalation maneuvers. Low resistance and repetitive use as per summary of product characteristics (SmPCs) were the reasons why we chose Trimbow® and Foster® pMDIs and Spiriva® Respimat® as patients need two consecutive inhalations twice and once daily, respectively, (28–30).

The availability of triple FDCs is growing, but clinicians are facing new challenges during the therapy of COPD patients. The effectiveness of FDC versus open triple therapy was previously investigated in the TRINITY clinical trial (31). Trimbow® pMDI was non-inferior regarding moderate-to-severe exacerbation rates and pre-dose FEV_1_ for week 52 versus the open triple therapy containing Foster® pMDI and a Spiriva® Handihaler® (31). Our study investigated a low resistance SMI as LAMA in open triple therapy and showed that it might produce higher PD in COPD patients independent of stable state or during AE. Consequently, using SMI LAMA might add to more effective therapy especially in patients who might profit from open triple therapy. Our results might suggest that in some cases clinicians can switch to a combination of pMDI and SMI as open triple therapy from triple FDC to increase pulmonary deposition of inhaled drugs in severe COPD patients. GOLD document is also suggesting adjustment of inhaled therapy by switching inhaler device or molecules within the same class. In COPD patients differences in effectivity regarding a given inhaled therapy were previously confirmed for LABA-LAMA combinations underlying the importance and possible benefit of switch (32).

Inhalation performance might be impaired despite the fact that these inhalers are suitable for patients with reduced lung function (33). Critical errors highly impact lung deposition. The higher intra-patient variability with different aerosol devices was described in previous studies often evaluating healthy volunteers or patients with different severity of airway obstruction (34, 35). In our setup patients used the simulation equipment with optimal and controlled technique as described by the manufacturer.

One of the most important disease deteriorating factor during the therapy of COPD is an episode of exacerbation. Exacerbation impairs function, breathing capacity and reduces inspiratory effort so patients are less able to perform sufficient and equal inhalation maneuvers and consequently have instable inhaled drug lung delivery. However, our study revealed that there was no significant difference between stable and exacerbated COPD patients using pMDI or SMI for PD values. Consequently, exacerbation does not considerably influence the effectivity of drug deposition and deposition repeatability for both devices. This can be explained that the use of inhaler techniques is regularly checked at our department patients with both stable and exacerbated COPD.

Several previous studies confirmed the impact of device handling on the effectiveness of inhaled medication. It is well known that the use of different inhalers or more than one device can negatively impact therapy (3, 15, 16). For COPD patients who are not able to generate adequate inspiratory effort, a pMDI or SMI device is recommended (36, 37). However, manufacturers design their device relying on the need to reach a satisfactory inspiratory flow, shortly a new parameter gains greater emphasis, the pressure drop during inhalation (38). It is important to note that our model did not use pressure drop values as input data. Despite that the number of different inhalers can worsen the effectiveness of inhalation therapy, our results suggest that the combination of an SMI and a pMDI can reach higher calculated drug PD compared to a FDC pMDI in individual patients.

COPD patients, especially those with severe disease, need proper education to acquire correct inhaler use. Achieving this goal, appropriate patient education is essential and regular assessment is needed during patient care (39, 40). Severe COPD patients are hospitalized frequently and during acute exacerbation device handling tend to be even more difficult. Our measurements revealed that an SMI performs evenly in patients with acute exacerbations. Repeatability highlights the importance of investigating measurement methods. Besides repeating different measurement on the same subjects by the same examiner, different measurement system can show varying result from same subjects, placing a greater focus on reproducibility (41).

Because many DPI devices cannot be used effectively with severe respiratory function impairment, greater emphasis should be placed on the use of low-resistance inhalers such as pMDI and SMI devices in patients with advanced COPD (42).

## 5. Conclusion

In summary in severe COPD patients using numerical deposition modeling lung deposition is higher for open triple combination using pMDI plus SMI device as compared to FDC pMDI. As each studied inhaled drug is dosed twice according SmPC for each device repeatability is of high interest. Around 40% of all COPD patients has >3% PD difference between the two inspiratory maneuvers, emphasizing the importance of optimal handling and teaching of devices. Important to note that there was no difference in PD between COPD patients during AE and S conditions. SMI repeatability seemed more robust in our study and might contribute to clinically meaningful difference in patients with persisting symptoms and exacerbation. By the latest recommendations, multiple devices are highly correlated to reduced adherence (43), therefore inhaler usage must be controlled on a regular basis. Further clinical studies and real-world data are needed to confirm the clinical effectiveness in this patient group.

## 6. Strengths and weaknesses of the study

Our study is the first comparing lung deposition values of a FDC pMDI and open triple therapy containing an SMI and pMDI in COPD patients. Numerical modeling provides us a more reproducible method to evaluate PD values without the burden of radiation. Weakness of our work is the low number of patients and repetitive maneuvers used for data input into the mathematical model.

## Data availability statement

The raw data supporting the conclusions of this article will be made available by the authors, without undue reservation.

## Ethics statement

The studies involving human participants were reviewed and approved by Semmelweis University Regional and Institutional Committee of Science and Research Ethics. The patients/participants provided their written informed consent to participate in this study.

## Author contributions

TE performed the through-device measurements and the calculations using numerical modeling. ZL coordinated the statistical analysis and conducted medical consultations. ÁF and PF ensured the use of the Stochastic Lung Model and provided scientific consultation. AN conducted scientific consultation. VM was responsible for the coordination and professional supervision of the investigation. All authors contributed to the article and approved the submitted version.

## Funding

This work was supported by KTIA_AIK_12_1_2012_ 0019 and the Research Grant of the Hungarian Respiratory Foundation (TE).

## Conflict of interest

VM received consultation fees from Astra Zeneca, Boehringer Ingelheim, Chiesi, Berlin Chemie Menarini, Orion Pharma, Novartis, GSK, Teva.

The remaining authors declare that the research was conducted in the absence of any commercial or financial relationships that could be construed as a potential conflict of interest.

## Publisher’s note

All claims expressed in this article are solely those of the authors and do not necessarily represent those of their affiliated organizations, or those of the publisher, the editors and the reviewers. Any product that may be evaluated in this article, or claim that may be made by its manufacturer, is not guaranteed or endorsed by the publisher.

## References

[1] GOLD, Global Strategy for Diagnosis, Management and Prevention of COPD. Available at: www.gold.copd.org, (2022).

[2] Asthma G.I.F., Global Strategy for Asthma Management and Prevention. Available at: www.ginasthma.org, (2022).

[3] Bosnic-Anticevich S Chrystyn H Costello R Dolovich MB Fletcher M Lavorini F. The use of multiple respiratory inhalers requiring different inhalation techniques has an adverse effect on COPD outcomes. Int J Chron Obstruct Pulmon Dis. (2017) 12:59–71. doi: 10.2147/COPD.S117196, PMID: 28053517 PMC5191843

[4] Usmani O Roche N Wahab E Israel S Jenkins M Trivedi R. A scintigraphy study of budesonide/glycopyrrolate/formoterol fumarate metered dose inhaler in patients with moderate-to-very severe chronic obstructive pulmonary disease. Respir Res. (2021) 22:261. doi: 10.1186/s12931-021-01813-w, PMID: 34620167 PMC8496011

[5] Wei X Hindle M Kaviratna A Huynh BK Delvadia RR Sandell D. In vitro tests for aerosol deposition. VI: realistic testing with different mouth-throat models and in vitro-in vivo correlations for a dry powder inhaler, metered dose inhaler, and soft mist inhaler. J Aerosol Med Pulm Drug Deliv. (2018) 31:358–71. doi: 10.1089/jamp.2018.1454, PMID: 29878859

[6] Schembri GP Miller AE Smart R. Radiation dosimetry and safety issues in the investigation of pulmonary embolism. Semin Nucl Med. (2010) 40:442–54. doi: 10.1053/j.semnuclmed.2010.07.007, PMID: 20920634

[7] Usmani OS Baldi S Warren S Panni I Girardello L Rony F. Lung deposition of inhaled Extrafine Beclomethasone Dipropionate/Formoterol Fumarate/Glycopyrronium bromide in healthy volunteers and Asthma: the STORM study. J Aerosol Med Pulm Drug Deliv. (2022) 35:179–85. doi: 10.1089/jamp.2021.0046, PMID: 35128939 PMC9416540

[8] Tomlinson HS Allen MD Corlett SA Chrystyn H. Comparison of urinary salbutamol 30 minutes post inhalation (USAL) and the methacholine dose to reduce the FEV1 by 20%(PD20) to identify the equivalence of inhaled salbutamol products. Eur Respir. (1999) 14:328s.

[9] Borgström L Nilsson M. A method for determination of the absolute pulmonary bioavailability of inhaled drugs: terbutaline. Pharm Res. (1990) 7:1068–70. doi: 10.1023/A:1015951402799 2281038

[10] Newnham DM McDevitt DG Lipworth BJ. Comparison of the extrapulmonary beta2-adrenoceptor responses and pharmacokinetics of salbutamol given by standard metered dose-inhaler and modified actuator device. Br J Clin Pharmacol. (1993) 36:445–50. doi: 10.1111/j.1365-2125.1993.tb00393.x, PMID: 12959292 PMC1364617

[11] Chrystyn H. Methods to identify drug deposition in the lungs following inhalation. Br J Clin Pharmacol. (2001) 51:289–99. doi: 10.1046/j.1365-2125.2001.01304.x, PMID: 11318763 PMC2014454

[12] Longest PW Bass K Dutta R Rani V Thomas ML el-Achwah A. Use of computational fluid dynamics deposition modeling in respiratory drug delivery. Expert Opin Drug Deliv. (2019) 16:7–26. doi: 10.1080/17425247.2019.1551875, PMID: 30463458 PMC6529297

[13] Koblinger L Hofmann W. Monte Carlo modeling of aerosol deposition in human lungs. Part I: simulation of particle transport in a stochastic lung structure. J Aerosol Sci. (1990) 21:661–74. doi: 10.1016/0021-8502(90)90121-D

[14] Farkas Á Jókay Á Balásházy I Füri P Müller V Tomisa G. Numerical simulation of emitted particle characteristics and airway deposition distribution of Symbicort® Turbuhaler® dry powder fixed combination aerosol drug. Eur J Pharm Sci. (2016) 93:371–9. doi: 10.1016/j.ejps.2016.08.036, PMID: 27552906

[15] Miravitlles M Soler-Cataluña JJ Alcázar B Viejo JL García-Río F. Factors affecting the selection of an inhaler device for COPD and the ideal device for different patient profiles. Results of EPOCA Delphi consensus. Pulm Pharmacol Ther. (2018) 48:97–103. doi: 10.1016/j.pupt.2017.10.006, PMID: 29031616

[16] Price D Keininger DL Viswanad B Gasser M Walda S Gutzwiller FS. Factors associated with appropriate inhaler use in patients with COPD - lessons from the REAL survey. Int J Chron Obstruct Pulmon Dis. (2018) 13:695–702. doi: 10.2147/COPD.S149404, PMID: 29520137 PMC5834182

[17] Barrons R Pegram A Borries A. Inhaler device selection: special considerations in elderly patients with chronic obstructive pulmonary disease. Am J Health Syst Pharm. (2011) 68:1221–32. doi: 10.2146/ajhp100452, PMID: 21690428

[18] Ghosh S Pleasants RA Ohar JA Donohue JF Drummond MB. Prevalence and factors associated with suboptimal peak inspiratory flow rates in COPD. Int J Chron Obstruct Pulmon Dis. (2019) 14:585–95. doi: 10.2147/COPD.S195438, PMID: 30880948 PMC6402615

[19] GOLD, Global Strategy for Diagnosis, Management and Prevention of COPD. Available at: www.goldcopd.org, (2015).

[20] Miller MR Hankinson JATS Brusasco V Burgos F Casaburi R Coates A. Standardisation of spirometry. Eur Respir J. (2005) 26:319–38. doi: 10.1183/09031936.05.00034805, PMID: 16055882

[21] Wanger J Clausen JL Coates A Pedersen OF Brusasco V Burgos F. Standardisation of the measurement of lung volumes. Eur Respir J. (2005) 26:511–22. doi: 10.1183/09031936.05.00035005, PMID: 16135736

[22] Erdelyi T Lazar Z Odler B Tamasi L Müller V. The repeatability of inspiration performance through different inhalers in patients with chronic obstructive pulmonary disease and control volunteers. J Aerosol Med Pulm Drug Deliv. (2020) 33:271–81. doi: 10.1089/jamp.2020.1594, PMID: 32460588 PMC7526298

[23] Horváth A Farkas Á Szipőcs A Tomisa G Szalai Z Gálffy G. Numerical simulation of the effect of inhalation parameters, gender, age and disease severity on the lung deposition of dry powder aerosol drugs emitted by Turbuhaler®, Breezhaler® and Genuair® in COPD patients. Eur J Pharm Sci. (2020) 154:105508. doi: 10.1016/j.ejps.2020.105508, PMID: 32836137

[24] Raabe O.G.Y. Schum Hsu-Chi Michael G. Phalen Robert F, Tracheobronchial Geometry: Human, Dog, Rat, Hamster. United States: Department of Commerce. (1976): p. 1–741.

[25] Haefeli-Bleuer B Weibel ER. Morphometry of the human pulmonary acinus. Anat Rec. (1988) 220:401–14. doi: 10.1002/ar.1092200410, PMID: 3382030

[26] Bland JM Altman DG. Statistical methods for assessing agreement between two methods of clinical measurement. Lancet. (1986) 1:307–10. PMID: 2868172

[27] Brand P Hederer B Austen G Dewberry H Meyer T. Higher lung deposition with Respimat soft mist inhaler than HFA-MDI in COPD patients with poor technique. Int J Chron Obstruct Pulmon Dis. (2008) 3:763–70. PMID: 19281091 PMC2650591

[28] EMC, Fostair 100/6 inhalation solution. (2007) Available at: https://www.medicines.org.uk/emc/product/6318/smpc.

[29] EMC, Spiriva Respimat 2.5 microgram, inhalation solution. (2017) Available at: https://www.medicines.org.uk/emc/product/407/smpc.

[30] EMA, Trimbow 87 micrograms/5 micrograms/9 micrograms pressurised inhalation, solution. (2017) Available at: https://www.ema.europa.eu/en/documents/product-information/trimbow-epar-product-information_en.pdf.

[31] Vestbo J Papi A Corradi M Blazhko V Montagna I Francisco C. Single inhaler extrafine triple therapy versus long-acting muscarinic antagonist therapy for chronic obstructive pulmonary disease (TRINITY): a double-blind, parallel group, randomised controlled trial. Lancet. (2017) 389:1919–29. doi: 10.1016/S0140-6736(17)30188-5, PMID: 28385353

[32] Feldman GJ Sousa AR Lipson DA Tombs L Barnes N Riley JH. Comparative efficacy of once-daily Umeclidinium/Vilanterol and Tiotropium/Olodaterol therapy in symptomatic chronic obstructive pulmonary disease: a randomized study. Adv Ther. (2017) 34:2518–33. doi: 10.1007/s12325-017-0626-4, PMID: 29094315 PMC5702366

[33] Ierodiakonou D Sifaki-Pistolla D Kampouraki M Poulorinakis I Papadokostakis P Gialamas I. Adherence to inhalers and comorbidities in COPD patients. A cross-sectional primary care study from Greece. BMC Pulm Med. (2020) 20:253. doi: 10.1186/s12890-020-01296-3, PMID: 32977779 PMC7519509

[34] Aswania O Ritson S Iqbal SM Bhatt J Rigby AS Everard ML. Intra-subject variability in lung dose in healthy volunteers using five conventional portable inhalers. J Aerosol Med. (2004) 17:231–8. doi: 10.1089/jam.2004.17.231, PMID: 15625815

[35] Fink JB Colice GL Hodder R. Inhaler devices for patients with COPD. COPD. (2013) 10:523–35. doi: 10.3109/15412555.2012.761960, PMID: 23537191

[36] Sanchis J Corrigan C Levy ML Viejo JL ADMIT Group. Inhaler devices – from theory to practice. Respir Med. (2013) 107:495–502. doi: 10.1016/j.rmed.2012.12.007, PMID: 23290591

[37] ERS In: Palange PRG, editor. Handbook of Respiratory Medicine. Sheffield, UK: The European Respiratory Society (2019). 886.

[38] Clark AR Weers JG Dhand R. The confusing world of dry powder inhalers: it is all about inspiratory pressures, not inspiratory flow rates. J Aerosol Med Pulm Drug Deliv. (2020) 33:1–11. doi: 10.1089/jamp.2019.1556, PMID: 31613682 PMC7041319

[39] Molimard M Raherison C Lignot S Balestra A Lamarque S Chartier A. Chronic obstructive pulmonary disease exacerbation and inhaler device handling: real-life assessment of 2935 patients. Eur Respir J. (2017) 49:1601794. doi: 10.1183/13993003.01794-2016, PMID: 28182569

[40] Gálffy G Mezei G Németh G Tamási L Müller V Selroos O. Inhaler competence and patient satisfaction with Easyhaler®: results of two real-life multicentre studies in asthma and COPD. Drugs R D. (2013) 13:215–22. doi: 10.1007/s40268-013-0027-3, PMID: 24043456 PMC3784057

[41] Alter P Orszag J Wouters EFM Vogelmeier CF Jörres RA. Differences in the measurement of functional residual capacity between body Plethysmographs of two manufacturers. Int J Chron Obstruct Pulmon Dis. (2022) 17:1477–82. doi: 10.2147/COPD.S363493, PMID: 35774592 PMC9239195

[42] Baloira A Abad A Fuster A García Rivero JL García-Sidro P Márquez-Martín E. Lung deposition and inspiratory flow rate in patients with chronic obstructive pulmonary disease using different inhalation devices: a systematic literature review and expert opinion. Int J Chron Obstruct Pulmon Dis. (2021) 16:1021–33. doi: 10.2147/COPD.S297980, PMID: 33907390 PMC8064620

[43] GOLD, Global Strategy for Diagnosis, Management and Prevention of COPD. (2023) Available at: www.gold.copd.org

